# Evaluation of the Threshold for an Improved Surface Water Extraction Index Using Optical Remote Sensing Data

**DOI:** 10.1155/2022/4894929

**Published:** 2022-03-04

**Authors:** Fajar Yulianto, Dony Kushardono, Syarif Budhiman, Gatot Nugroho, Galdita Aruba Chulafak, Esthi Kurnia Dewi, Anjar Ilham Pambudi

**Affiliations:** Remote Sensing Research Center, Aeronautics and Space Research Organization, National Research and Innovation Agency (BRIN), Jl. Kalisari No. 8, Pekayon, Pasar Rebo, Jakarta 13710, Indonesia

## Abstract

In this study, we proposed an automatic water extraction index (AWEI) threshold improvement model that can be used to detect lake surface water based on optical remote sensing data. An annual Landsat 8 mosaic was created using the Google Earth Engine (GEE) platform to obtain cloud-free satellite image data. The challenge of this study was to determine the threshold value, which is essential to show the boundary between water and nonwater. The AWEI was selected for the study to address this challenge. The AWEI approach was developed by adding a threshold water value based on the split-based approach (SBA) calculation analysis for Landsat 8 satellite images. The SBA was used to determine local threshold variations in data scenes that were used to classify water and nonwater. The class threshold between water and nonwater in each selected subscene image can be determined based on the calculation of class intervals generated by geostatistical analysis, initially referred to as smart quantiles. It was used to determine the class separation between water and nonwater in the resulting subscene images. The objectives of this study were (a) to increase the accuracy of automatic lake surface water detection by improvising the determination of threshold values based on analysis and calculations using the SBA and (b) to conduct a test case study of AWEI threshold improvement on several lakes' surface water, which has a variety of different or heterogeneous characteristics. The results show that the threshold value obtained based on the smart quantile calculation from the natural break approach (AWEI ≥ −0.23) gave an overall accuracy of close to 100%. Those results were better than the normal threshold (AWEI ≥ 0.00), with an overall accuracy of 98%. It shows that there has been an increase of 2% in the accuracy based on the confusion matrix calculation. In addition to that, the results obtained when classifying water and nonwater classes for the different national priority lakes in Indonesia vary in overall accuracy from 94% to 100%.

## 1. Introduction

Remote sensing plays a role in providing information on large-scale monitoring of surface waters, with the advantages of high spatiotemporal resolution, multisensors, and near-real-time operational data [1–3]. Monitoring can be performed faster when compared with direct measurements in the field and can assist in strategic planning to cost reduction measures, with limited human and scientific resources [4, 5]. Based on a review of optical remote sensing, several methods can be used to extract surface water. Several studies have also developed more effective surface water extraction methods using water index methods [6]. McFeeters [7] developed the normalized difference water index (NDWI) based on the green and near-infrared (NIR) bands to delineate features of open water by using a threshold value greater than zero as a delimiter for extracting surface water. This means that the positive values are classified as water and negative values are classified as nonwater. The NDWI approach was modified by Xu [8] to become the modified normalized difference water index (MNDWI) by replacing the NIR band with the shortwave-infrared (SWIR) band. This was done because the application of NDWI in some water areas adjacent to built-up land produces noise extraction of water information mixed with the built-up land. To eliminate the noise in the water extraction results, the NDWI was modified by Xu [8] into MNDWI.

Furthermore, Feyisa et al. [9] developed the AWEI using a multiple-band index formulation based on four-band satellite imagery, namely green, NIR, SWIR, and mid-infrared (MIR) bands. This approach was developed to improve the extraction results of water information from several previous water indices, which was insufficient to use only two satellite imagery bands to achieve high accuracy. This was done because noise cannot distinguish between water and darker pixel (nonwater) surfaces, such as the shadow. Implementation of several indices of such water has been used in various other aspects, such as analysis and flood monitoring [10–12], environment analysis [13], water surface mapping [14–16], water-quality assessment [17], and agriculture [18].

Based on a review of several water indices, the AWEI was selected for this study because it gives better results in representing available surface water. In general, the results of the water index calculation require a threshold value to separate the water and nonwater objects. Based on the results of a review of several previous studies, it was shown that the threshold for the water class is a positive value, which means it can separate between required water and nonwater threshold values greater than 0 (null) [6, 9, 17, 19]. Determining the threshold value plays an essential role in showing the boundary between water and nonwater, which was a challenge in this study. The split-based approach (SBA) was initially proposed by Bovolo and Bruzzone [20] and is used to determine local threshold variations in data scenes; it was used to classify water and nonwater classes in this study. It was also chosen to improve the global threshold limitations in these objects. The SBA was used by Bovolo and Bruzzone [20] to identify the impact of land changes due to the tsunami disaster, applied to multitemporal imagery.

To address this challenge, one of the index approaches to water, AWEI, was selected and used in this study. The AWEI approach was developed by adding a threshold water value based on the SBA calculation analysis for images applied to Landsat 8 satellite imagery. Furthermore, the same approach was used by Martinis et al. [21] for flood detection with high-resolution TerraSAR-X data and Yulianto et al. [22] to determine threshold flood detection with ALOS PALSAR data.

This study used the AWEI, a multiple-band index, as an automatic approach for lake surface water extraction. Furthermore, the AWEI was developed with the aim of (a) increasing the accuracy of automatic lake surface water mapping by improvising the determination of threshold values based on analysis and calculations using the SBA and (b) conducting test case studies of AWEI threshold improvement results on the surface water of several lakes, which has a variety of different or heterogeneous characteristics.

## 2. Materials and Methods

### 2.1. Study Area

**Figure 1:**
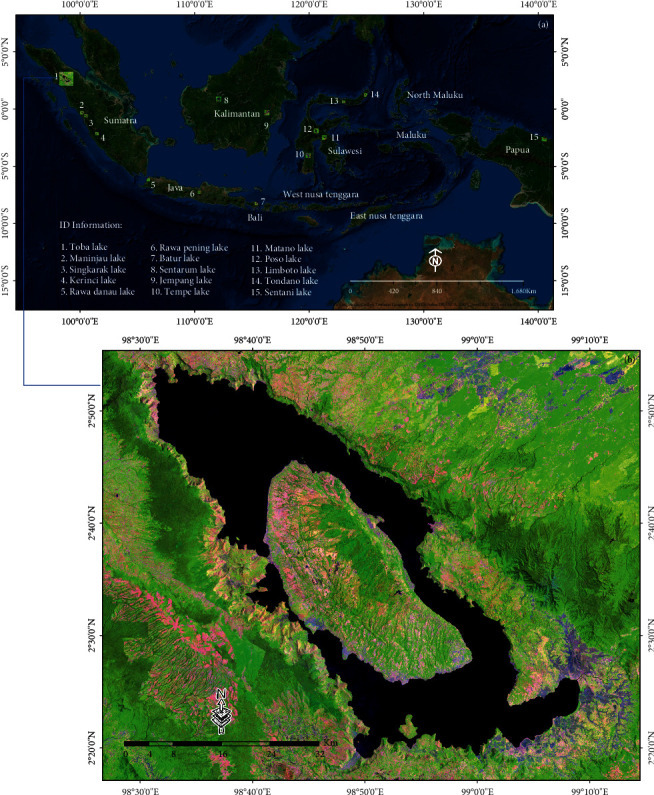
(a) Location of the 15 national priority lakes in Indonesia for management and spatial planning. At these locations, a test case study of AWEI threshold improvement was conducted, which represented the characteristics of other lakes. (b) Toba Lake, located in North Sumatra Province, Indonesia, was used as a test case study area to develop an AWEI threshold improvement model to detect lake surface water on remotely sensed optical data.

Toba Lake, also known as *Danau Toba*, which is located in North Sumatra, Indonesia, at coordinates 2.35°–2.88° North and 98.52°–99.1° East (Figure 1), was used as a test case study area to develop an AWEI threshold improvement model to detect lake surface water on remotely sensed optical data that represent the characteristics of the type of volcanic-tectonic lake [23, 24]. The AWEI threshold improvement to detect lake surface water was tested at several other locations representing different lake characteristics in Indonesia. The Ministry of Environment of the Republic of Indonesia (KLHK) states that 15 national priority lakes in Indonesia need to be saved to restore, preserve, and maintain lake functions based on the principle of ecosystem to balance the environment's carrying capacity. The 15 lakes (including Toba Lake) can be classified based on the characteristics of the process of formation, namely (a) the techno-volcanic process, comprising Toba Lake (North Sumatra); (b) the tectonic process, including Poso Lake (South East Sulawesi), Tempe Lake (South Sulawesi), Tondano Lake (North Sulawesi), Singkarak Lake (West Sumatra), Limboto Lake (Gorontalo), and Sentani Lake (Papua); (c) the volcanic process, including Batur Lake (Bali), Kerinci Lake (Jambi), Maninjau Lake (West Sumatra), Matano Lake (South Sulawesi), Rawa Pening Lake (Central Java), and Rawa Dano (Banten); and (d) the flood plain process, including Sentarum Lake (West Kalimantan) and Jempang Lake (East Kalimantan).

### 2.2. Data Set

The main problem in using optical satellite imagery data is the obscuring of objects on the surface of the Earth by clouds. This can be solved by creating cloud-free satellite imagery data annually. In this study, such creation from Landsat 8 was performed using the Google Earth Engine (GEE) platform. Input data were obtained based on Landsat 8 Surface Reflectance Tier 1 data collection. This data set comprises the atmospherically corrected surface reflectance, which is based on the Landsat Ecosystem Disturbance Adaptive Processing System (LaRSC), and the various stages of the process consisting of cloud, shadow, water, and snow mask are produced using CFMASK [25–27]. Filter dates are needed to determine the date range selection time limit to get annual Landsat 8 in 2019. In this case, the date used was 01 January 2019 to 31 December 2019. Furthermore, the determination of the area of interest (AOI) boundary for the lake surface water refers to digital vector data published by the Ministry of Environment and Forestry, Indonesia (KLHK), based on the 15 national priority lakes.

### 2.3. AWEI Approach

The automatic water extraction index (AWEI) approach was used as the water index by removing shadow pixels, which can be formulated based on [9]

(1) $$\begin{array}{c} \text{AWEI}=4x\left(G-\text{MIR}\right)-\left(0.25x\text{NIR}+2.75x\text{SWIR}\right), \end{array}$$

where AWEI is the automatic water extraction index; *G* is the green channel, with wavelength 0.53–0.59 *µ*m; NIR is the near-infrared channel, with wavelength 0.85–0.88 *µ*m; SWIR is the shortwave-infrared channel, with wavelength 1.57–1.65 *µ*m; and MIR is the mid-infrared channel, with wavelength 2.11–2.29 *µ*m, from the Landsat 8 imagery.

### 2.4. Split-Based Approach (SBA)

#### 2.4.1. Image Tiling and Split Selection

**Figure 2:**
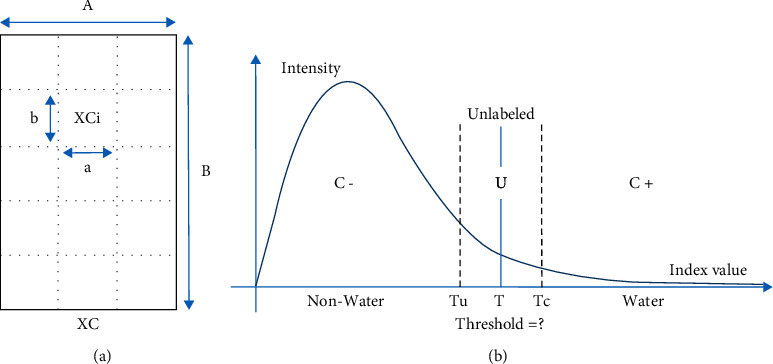
(a) Illustration of the SBA for scene image XC with size *A* *x* *B* and subscene image XCi with size *a* *x* *b*. (b) Graphical illustration of the distribution of pixel values for the two classes water (*C*+) and nonwater (*C*−), and unlabeled class (*U*) for which the threshold value (*T*=?) is needed as a boundary between the water and nonwater classes.

The split-based approach (SBA) was used to determine local threshold variations in the scene data used to classify “water” and “nonwater” in the study and improve the global limitation threshold separating the two classes. The SBA is illustrated in Figure 2, which illustrates a scene image XC that has dimensions *A* *x* *B* and a split scene image XCi (subscene image) with dimensions *a* *x* *b*. *A* *x* *B* is a grid cell in one scene image, while *A* is a line in scene image *A* *x* *B*. *B* is a column in scene image *A* *x* *B*; XCi is the subscene image XC to *i*, where (*i*=1,  2,  3,   …, *n*); *a* is the line of the split parts of the subscene image XCi, while *b* is the column of the split parts of subscene image XCi [20, 22].

In this study, the result of the AWEI image is used to implement the SBA with size *A* *x* *B*. The split scene for the result of the AWEI is created using the grid index features (GIF), with image size 2 km × 2 km, so that it could be obtained on each XCi with size *a*=2km and *b*=2km. A total of 1677 split scene images were used in the study to calculate the local statistics parameters for each split scene image; in this case, XCi, where *i*=1,  2,  3,   …, *n*. The statistical parameters used in calculating each split scene image were the minimum, maximum, mean, and standard deviation values.

The coefficient of variation value (CVXCi) was formulated as the ratio between the mean (*µ*XCi) and standard deviation (*σ*XCi) for the split scene image (XCi) was used in an appropriate statistical measure to select split images within the data range. The scene ratio (RXCi) was formulated as the ratio between the mean (*µ*XCi) for the split scene image and the global mean (*µ*XC) for one scene image. Furthermore, the distribution of measurement results, the level of data variation, and the probability that the splits contained more than one semantic class were determined. The measurement results of the coefficient of variation values of each split scene image can be plotted on the *X*-axis, and the scene ratio values of each split scene image can be plotted on the *Y*-axis.

(2) XCi′=XCi|CVXCi≥0^RXCi0.5≤…≤1.0, Ci=1,2,3,…,n,

(3) $$\begin{array}{c} {\text{XCi}}^{\prime \prime }=\;\left\{{\text{XC}1}^{\prime },\text{XC}2^{\prime },\ldots ,{\text{XCi}}^{\prime }|\Delta \text{CVXC}1^{\prime }\leq \Delta \text{CVXC}2^{\prime }\leq \ldots \leq \Delta {\text{CVXCi}}^{\prime },\;n\leq {\text{XC}}^{\prime \prime }\right\}. \end{array}$$

The selection of the first-stage split scene image in the scene data (XC′) of subscene images (XCi′) was made by investigating several data sets to show the optimal representation of water and nonwater, based on equation (2). Furthermore, the selection of the second-stage split scene image in the scene data (XCi^″^) of subscene images (XCi^″^) in equation (3) was made from the data set selected from (XCi′), (XCi′), and used to determine the calculation of the local threshold in the data set, selected and based on the Euclidean distance calculation (ΔCVXCi′). These technical procedures refer to Martinis et al. [21].

#### 2.4.2. Automatic Threshold Selection Procedure

**Figure 3:**
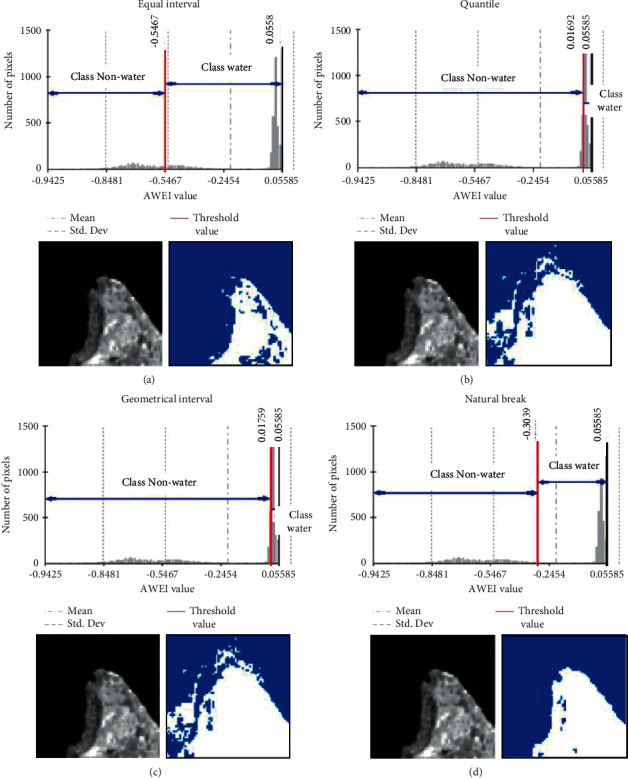
Automatic threshold selection procedure using geostatistical analysis approach: (a) equal interval, (b) quantile, (c) geometrical interval, and (d) natural break (an example for split scene ID: AWEI_15).

The geostatistical analysis approach initially referred to as smart quantiles was used to help determine the class separation between water and nonwater in the resulting subscene images selected (XCi^″^). Equal interval, quantile, geometrical interval, and natural break comprised the classification approach based on the intervals and statistical data distribution used in this study.  Figure 3 shows the automatic threshold selection procedure using the geostatistical analysis approach: (a) equal interval, (b) quantile, (c) geometrical interval, and (d) natural break (an example for split scene ID: AWEI_15). The class threshold between water and nonwater in each selected subscene image can be determined based on the calculation of class intervals generated by each geostatistical analysis method. Furthermore, the average value of each local threshold generated from all the subscene images selected was used as the final threshold to determine the class boundary between water and nonwater in one overall image scene (XC).

Equal interval classes can be grouped across a range of values that allow them to be divided into equal-sized intervals. Usually, there are fewer endpoints at the extreme, and the number of values in the extreme class is lower. The quantile class can be grouped based on a range of values that allow it to be divided into intervals of unequal size so that the number of values is the same in each class. The classes at the extreme and middle have the same number of values. The geometric intervals class can be grouped based on a classification scheme by creating class breaks based on class intervals that have a geometric sequence. The geometric coefficient can change once (in reverse) to optimize the class range. The algorithm creates geometric intervals by minimizing the sum of the squares of the number of elements in each class. This ensures that each class range has approximately the same number of values as each class and that changes between intervals are relatively consistent. The natural break (Jenks) class can be grouped based on natural groupings inherent in the data. Class breaks are created in the best way possible, grouping the same values and maximizing class differences. These features are divided into defined classes in which there is a relatively significant difference in data values [28].

### 2.5. AWEI Threshold Improvements

The calculation of the local threshold results was based on the second-stage split scene image selection in the scene data (XC^″^) of the subscene images (XCi^″^), (XCi^″^), using the equal interval, quantile, geometrical interval, and natural break approach that were used to determine the AWEI threshold improvements. A total of 20 selected subscene images (XCi^″^) were used to determine the local threshold value. The local threshold value obtained for each subscene image (20 subscene images) was calculated for the minimum, maximum, mean, and standard deviation values. Furthermore, the process to obtain the global threshold value was determined by calculating the mean value minus the standard deviation (the value of the mean − standard deviation of the selected 20 subscene images).

### 2.6. Accuracy Assessment of the AWEI Threshold Improvements

Evaluation of the reliability level and accuracy assessment of the AWEI threshold improvement was made using the visual and statistical approaches. The visual approach was performed to compare the appearance of objects in the Landsat 8 image and the classification results of water and nonwater classes generated by the thresholds of the equal interval, quantile, geometrical interval, and natural break. Meanwhile, the statistical approach to the confusion matrix calculation considers commission error, omission error, user accuracy, producer accuracy, total error, kappa, and overall accuracy. It was used to evaluate the class's quality generated by the thresholds of the equal interval, quantile, geometrical interval, and natural break and compared based on map references [29–31]. In this study, the map references were obtained based on the on-screen visual digitization process in Landsat 8 imagery, which was performed at several lake locations that are national priorities in Indonesia.

## 3. Results

### 3.1. Image Tiling and Split Selection from the SBA

**Figure 4:**
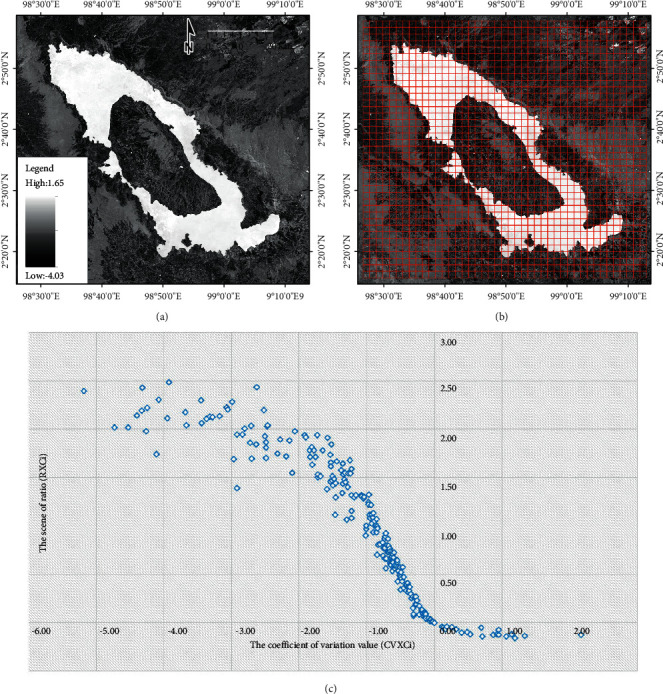
(a) Result calculation of the AWEI approach for (a) scene image and (b) split scene image with GIF at the size of 2 km × 2 km; a total of 1677 split scene images were used in the study to calculate the local statistics parameters for each split scene image. (c) Measurement results of the coefficient of variation value of each split scene image plotted on the *X*-axis and the scene ratio value plotted on the *Y*-axis.

**Figure 5:**
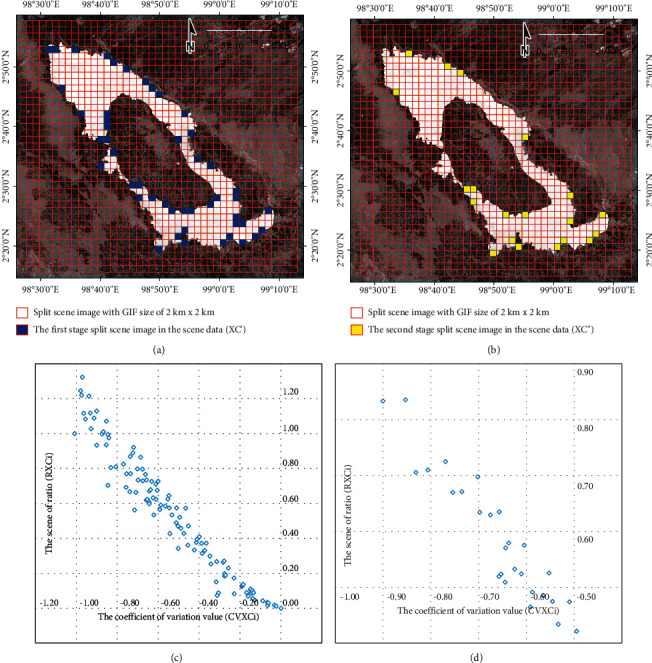
(a) Location of the split scene image with GIF was at a size of 2 km × 2 km for the first-stage split scene image in the scene image data (**XCi**′). (b) Location of the split scene image with GIF for the second-stage split scene image in the scene image data (**XCi**^″^). (c) The coefficient of variation value on the first-stage split scene image can be plotted on the *X*-axis, and the scene ratio value can be plotted on the *Y*-axis. (d) Plot of the second-stage split scene image for the coefficient of variation value on the *X*-axis and the ratio of the scene value on the *Y*-axis.

The result calculation of the AWEI approach for scene images or split scenes from the SBA is presented in Figure 4. A total of 1677 split scene images were used in this study to calculate local statistics parameters (the coefficient of variation value and the scene ratio value) for each split scene image, with GIF conducted at a size of 2 km × 2 km. In addition, the results of split scene image location for the first-stage split scene image in the scene image data (XCi′) and also for the second-stage split scene image in the scene image data (XCi′) are presented in Figure 5.

### 3.2. Automatic Threshold Selection Procedure

**Figure 6:**
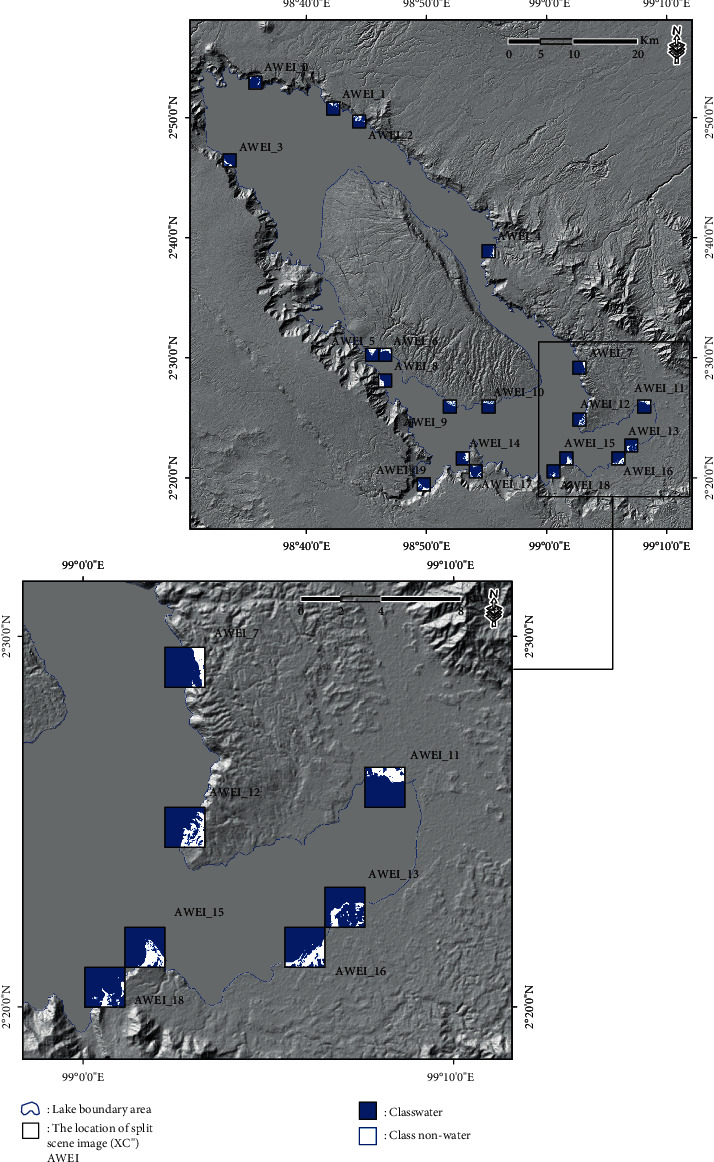
Location of split scene image selection in the scene data (**XC**^″^) of the subscene images (**XCi**^″^) with a size of 2 km × 2 km based on the equal interval threshold results.

**Figure 7:**
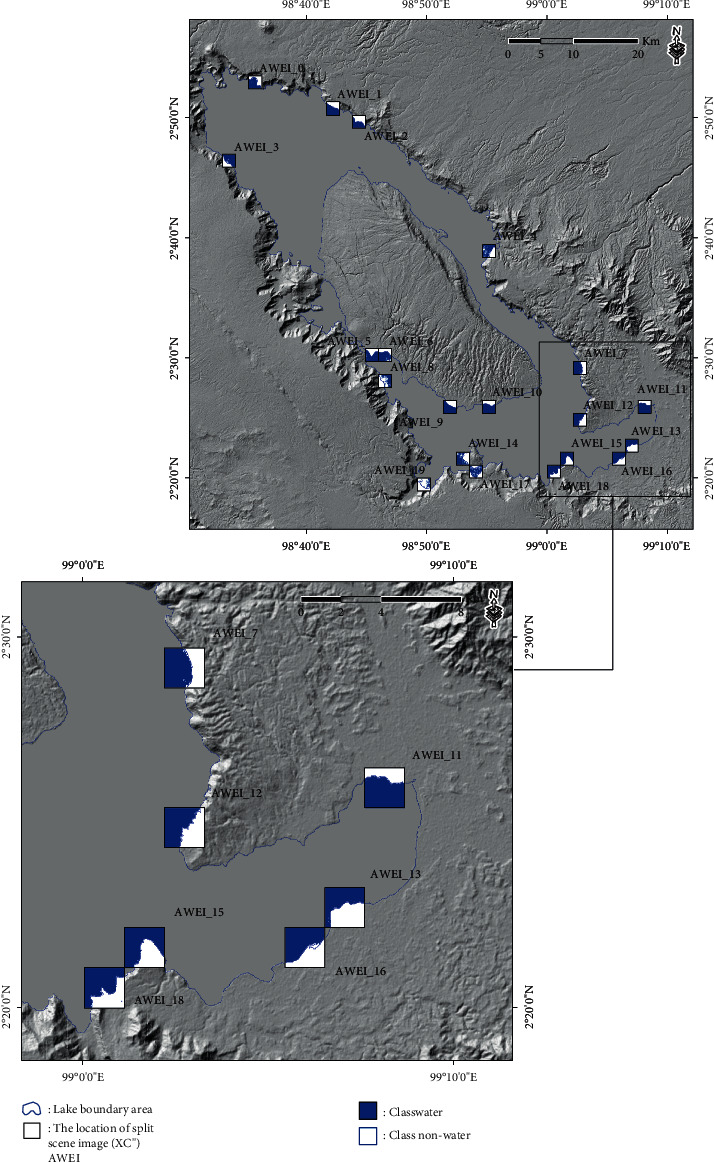
Location of the split scene image selection in the scene data (**XC**^″^) of the subscene images (**XCi**^″^) with a size of 2 km × 2 km based on the quantile threshold results.

**Figure 8:**
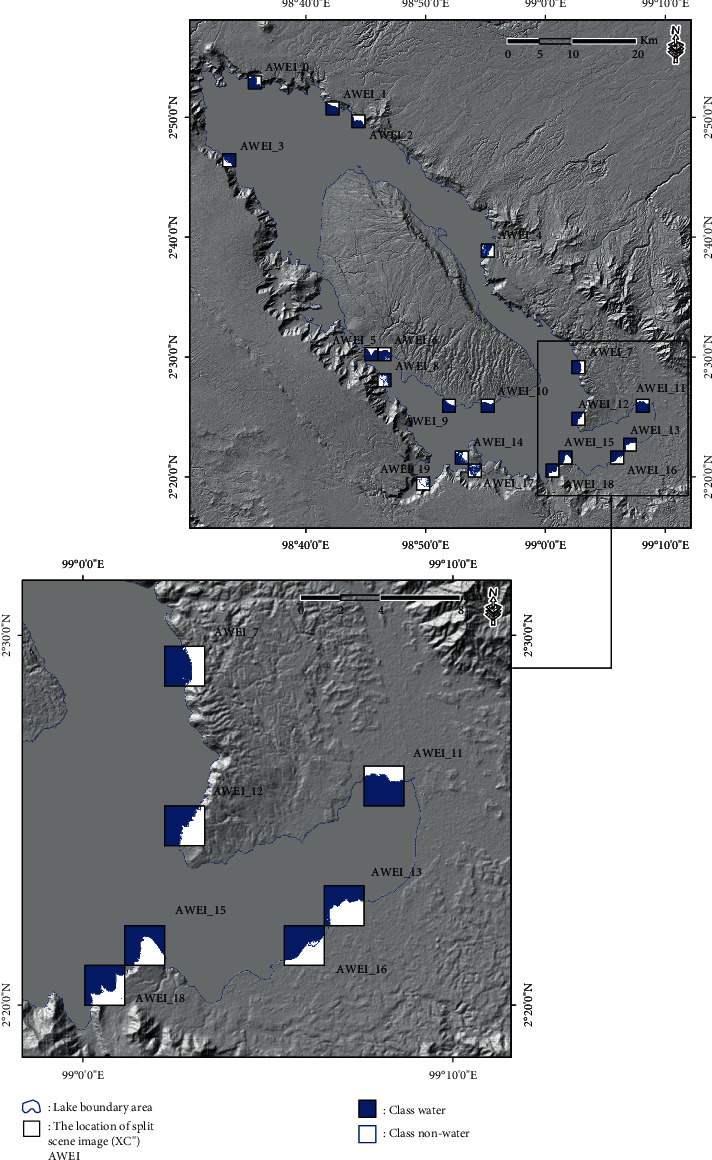
Location of the split scene image selection in the scene data (**XC**^″^) of the subscene images (**XCi**^″^) with a size of 2 km × 2 km based on the geometrical interval threshold results.

**Figure 9:**
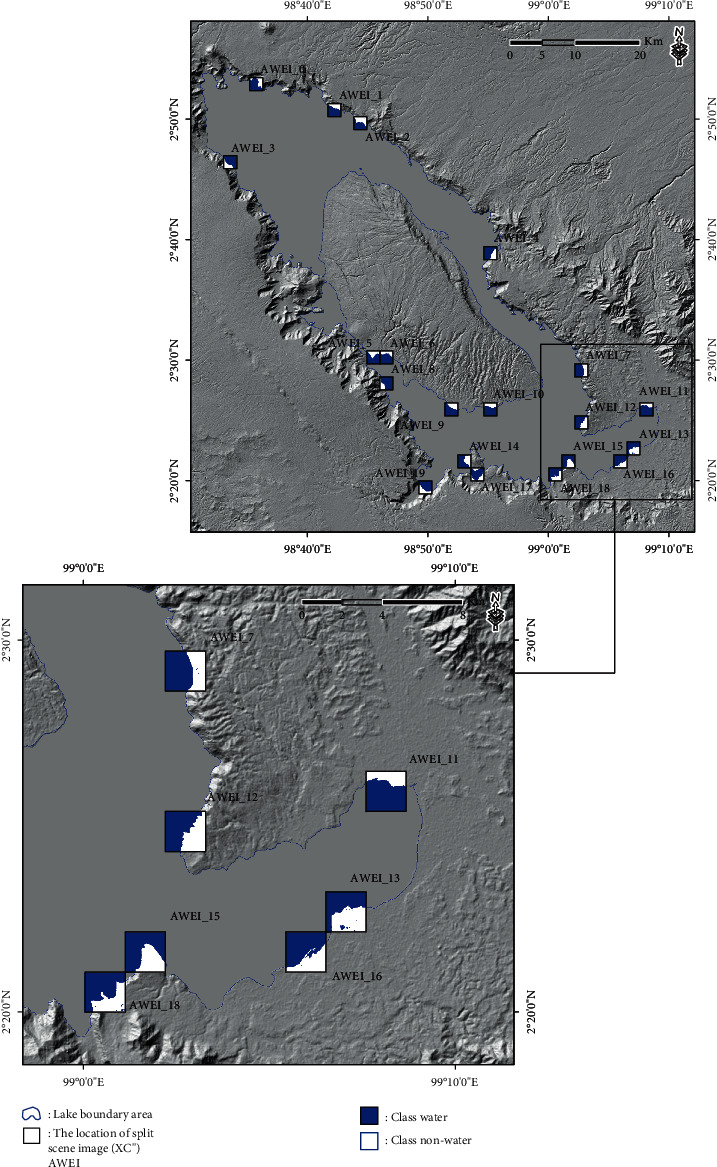
Location of the split scene image selection in the scene data (**XC**^″^) of the subscene images (**XCi**^″^) with a size of 2 km × 2 km based on the natural break threshold results.

The automatic threshold selection procedure was performed with the geostatistical analysis approach to determine the class separation between water and nonwater in the resulting subscene images selected (XCi′). The result calculation of the local threshold based on the second-stage split scene image selection in the scene data (XC^″^) of the subscene images (XCi′) is presented in Table 1. In addition, the results of the class separation between water and nonwater in the split scene image selection based on the results of the equal interval, quantile, geometrical interval, and natural break threshold are presented in Figures 6–9.

### 3.3. AWEI Threshold Improvements

**Figure 10:**
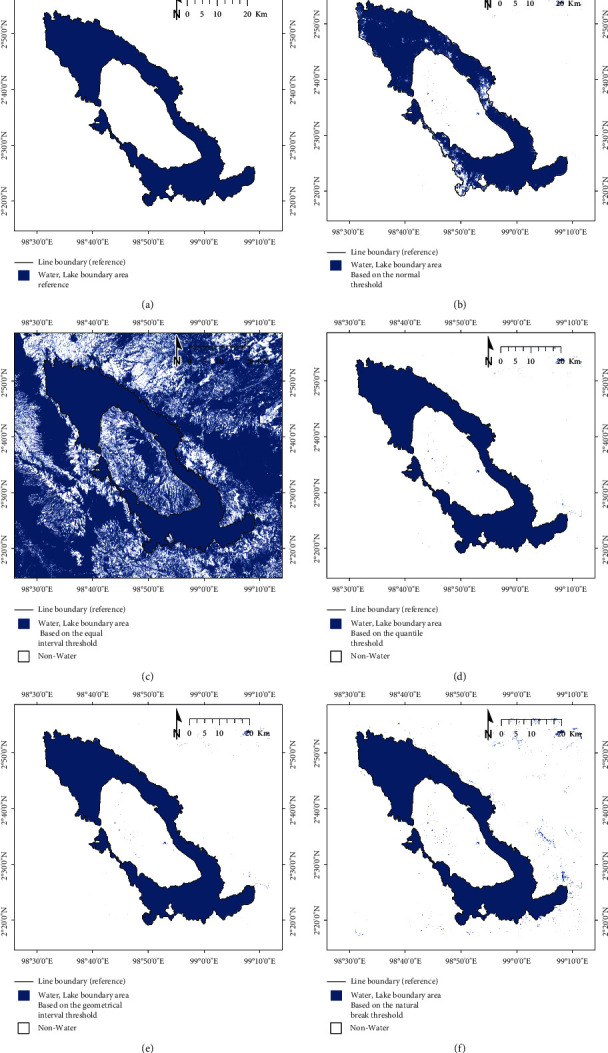
Test case results of the AWEI threshold improvement to detect lake surface water (water and nonwater class) in Toba lake: (a) map references were obtained based on the on-screen visual digitization of Landsat 8 imagery from 2019; (b) classification based on the normal threshold; (c) classification based on the equal interval threshold; (d) classification based on the quantile threshold; (e) classification based on the geometrical interval threshold; and (f) classification based on the natural break threshold.

The local threshold result calculation based on the second-stage split scene image selection using the equal interval, quantile, geometrical interval, and natural break approach has been used to determine the AWEI threshold improvements. The local threshold values obtained for the 20 subscene images were calculated for the minimum, maximum, mean, and standard deviation values (Table 1). The AWEI threshold improvement to obtain the global threshold value was determined by calculating the mean value minus the standard deviation, based on the 20 subscene images selected, with the results presented in Table 2. Furthermore, the AWEI threshold improvement test case results to detect lake surface water in Toba lake, based on the comparison of normal, equal interval, quantile, geometrical interval, and natural break thresholds, are presented in Figure 10.

### 3.4. Accuracy Assessment of the AWEI Threshold Improvements

**Figure 11:**
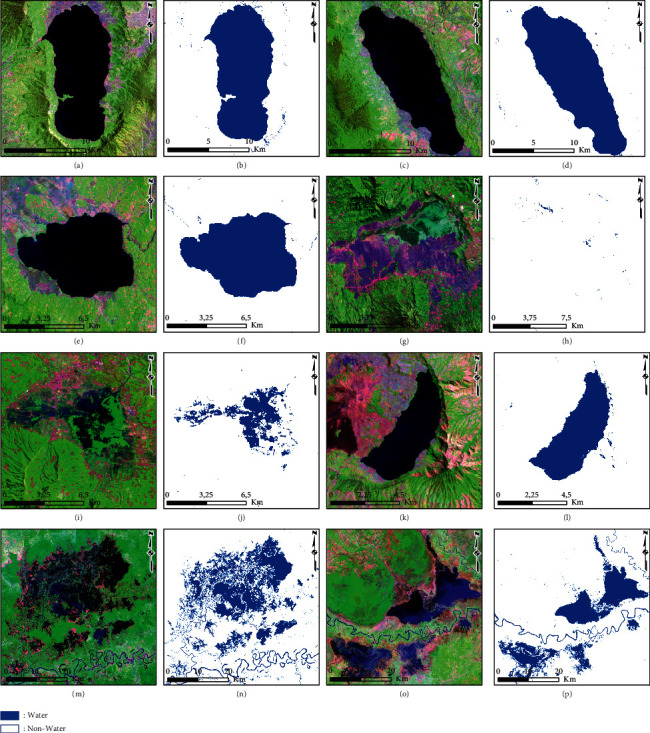
Results of water and nonwater classification based on the natural break threshold (AWEI ≥ −0.232): (a, b) Maninjau Lake; (c, d) Singkarak Lake; (e, f) Kerinci Lake; (g, h) Rawa Danau; (i, j) Rawa Pening Lake; (k, l) Batur Lake; (m, n) Sentarum Lake; and (o, p) Jempang Lake.

**Figure 12:**
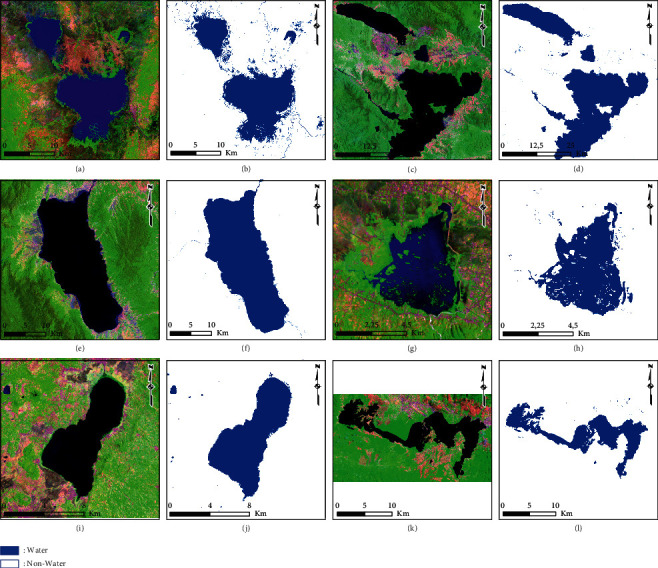
Results of water and nonwater classification based on the natural break threshold (AWEI ≥ −0.232): (a, b) Tempe Lake; (c, d) Matano Lake; (e, f) Poso Lake; (g, h) Limboto Lake; (i, j) Tondano Lake; and (k, l) Sentani Lake.

The results of the summary of the classification accuracy assessment of the AWEI threshold improvement are presented in Table 3. A statistical assessment approach based on confusion matrix calculation was used to account for commission error, omission error, user accuracy, producer accuracy, total error, kappa, and overall accuracy. The accuracy calculations show that the threshold value derived from natural break had the best overall accuracy of 99.86%, while that derived from equal interval had the lowest overall accuracy of 49.38%. Furthermore, the best results from determining the natural break threshold value were implemented to classify lake surface water (water and nonwater classes) at several other priority lake locations in Indonesia; these can also be compared with the results of the lake surface water classification derived from the normal threshold. The comparison results of the lake surface water classification from the natural break and normal thresholds are presented in Figures 11 and 12. In addition, the results of the calculation using the confusion matrix statistical approach are presented in Table 4.

## 4. Discussion

This research was conducted to contribute to the efforts to improve accuracy in detecting and rapidly classifying lake surface water based on optical remote sensing data in the Indonesian territory. In addition, the results can also be used to perform several other surface water mappings and analyze surface water changes caused by environmental dynamics. The SBA was applied in this study to determine and obtain statistical variations in local pixel values, which were applied to optical satellite imagery from Landsat 8 using the AWEI as the water index. Furthermore, variations in local pixel values from the AWEI were used as input to determine the global threshold in classifying water and nonwater objects in the study area. Based on research conducted by Feyisa et al. [9], the AWEI is a development of the previous water indexes. It has a better accuracy in detecting various types of surface water in various environmental conditions than the NDWI developed by McFeeters [7] and the MNDWI developed by Xu [8].

Determining the threshold value is essential in classifying water and nonwater classes. In general, several studies have stated that a positive value for the AWEI or an AWEI value of more than 0 is the boundary between water and nonwater classes [6, 17, 19]. According to Feyisa et al. [9], many mappings related to surface water and increasing its accuracy have been presented in related studies. However, there are limitations in making judgments regarding accuracy at a more detailed pixel level (subpixel) in practice. In this study, make tiling and split image selection from the SBA aimed to establish the variation in pixel values locally, with GIF at a size of 2 km × 2 km. A total of 1677 split scene images were used to calculate local statistical parameters (the coefficient of variation and the scene ratio value), wherein 20 selected subscene images were used as location tiles in the study area, representing local pixel value variations to determine the threshold value for water and nonwater classes.

Geostatistical analysis approaches consisting of the equal interval, quantile, geometrical interval, and natural break were used to automatically determine the threshold value for 20 selected subscene images divided into two classes (water and nonwater) and calculated based on the overall mean value − standard deviation. The results of calculating accuracy using the confusion matrix show that the threshold value generated from the natural break approach (AWEI ≥ −0.232) has the best accuracy, with an overall accuracy of 99.86% (as shown in Table 2 and Figure 10). This approach also has a better accuracy when compared with the normal threshold condition (AWEI ≥ 0), whose overall accuracy is 98.17%. This shows that there is an increase of 1.69% in accuracy from the threshold improvement that has been proposed and tested in this study. The threshold value derived from geometrical intervals (AWEI ≥ −0.084) and quantile (AWEI ≥ −0.089) has the same overall accuracy of 99.84%, which shows an increase of 1.67% in accuracy from the normal threshold value (AWEI ≥ 0). However, the water and nonwater classification results derived from the calculation of the threshold equal interval (AWEI ≥ −0.676) have an accuracy of 49.38%.

To study further about its more comprehensive application, the best threshold improvement value (natural break on AWEI ≥ −0.232) was applied to several national priority lake locations in Indonesia. The implementation was tested on several lakes, which have different process formation characteristics, which can be grouped based on (a) the techno-volcanic process, as with Toba Lake (North Sumatra); (b) the tectonic process, including Poso Lake (South East Sulawesi), Tempe Lake (South Sulawesi), Tondano Lake (North Sulawesi), Singkarak Lake (West Sumatra), Limboto Lake (Gorontalo), and Sentani Lake (Papua); (c) the volcanic process, including Batur Lake (Bali), Kerinci Lake (Jambi), Maninjau Lake (West Sumatra), Matano Lake (South Sulawesi), Rawa Pening Lake (Central Java), and Rawa Dano (Banten); and (d) the flood plain process, including Sentarum Lake (West Kalimantan) and Jempang Lake (East Kalimantan). The results show that using the natural break threshold implemented on several national priority lakes in Indonesia to classify water and nonwater classes had a variation in overall accuracy ranging from 93.58% to 99.59%.

This study applied AWEI threshold improvement to cloud-free Landsat 8 satellite imagery data, which displays the median pixel value based on the filter date data used as input. This will affect the quality and accuracy if the development of the AWEI threshold is applied to a Landsat 8 image with one recording date (single date), which is also influenced by other factors such as the use of its atmospheric correction type. The study has not considered the influence of seasonal variations on selecting the filter date data used as input in creating the cloud-free Landsat 8 satellite imagery data. This will be a challenge for future research to be more specific in considering variations in seasonal conditions and their development for other types of optical satellite image sensors. In addition, there are several limitations in applying the study results in detecting lake surface water caused by the covering of the surface of the water body by a vegetation canopy above it. This can be exemplified at the Lake Rawa Danau (Banten Province) location, visually presented in Figure 11(g), with the classification results in Figure 11(h).

## 5. Conclusion

The objectives of this study were to increase the accuracy of automatic lake surface water classification using an AWEI based on analysis and calculations with the SBA and to determine the threshold value in classifying water and nonwater classes and conduct a test case study of AWEI threshold improvement results on the surface water of several lakes with a variety of different or heterogeneous characteristics. The SBA was used to perform split image selection in determining variations in the local pixel value of the AWEI, which represents the study area based on statistical calculations. Furthermore, the split image selection results were used to determine the threshold value in classifying water and nonwater classes. The results show that there was an increase of 1.69% in accuracy in the classification of water and nonwater classes based on the natural break approach (AWEI ≥ −0.232) compared with the results of normal threshold conditions (AWEI ≥ 0), with a comparison of the overall accuracy for a natural break and normal thresholds of 99.86% and 98.17%, respectively. The threshold value results derived from the natural break approach (AWEI ≥ −0.232) were used as the threshold improvement in this study and applied to several national priority lakes in Indonesia that have different formation processes. The results also can be applied to the monitoring of multitemporal lake water conditions at medium resolution and to support mapping efforts at a scale of 1 : 50,000–1 : 100,000 in Indonesia. Further development is needed in future research using other types of optical satellite image sensors and on more specific seasonal variations so that the temporal density variations in monitoring the dynamics of changes in lake surface water can be increased.

## Figures and Tables

**Table 1:** Result calculation of the local thresholds based on the second-stage split scene image selection in the scene data (**XC**^″^) of the subscene images (**XCi**^″^) using the equal interval, quantile, geometrical interval, and natural break approach.

Split scene ID	Threshold value			
	Equal interval	Quantile	Geometrical interval	Natural break
AWEI_0	−0.430	0.010	0.016	−0.155
AWEI_1	−0.466	0.040	0.041	−0.160
AWEI_2	−0.577	−0.331	−0.309	−0.184
AWEI_3	−0.447	0.025	0.027	−0.195
AWEI_4	−0.596	−0.007	−0.009	−0.198
AWEI_5	−0.592	0.013	0.013	−0.232
AWEI_6	−0.660	0.008	0.004	−0.216
AWEI_7	−0.508	0.016	0.016	−0.195
AWEI_8	−0.502	−0.018	−0.017	−0.263
AWEI_9	−0.438	0.050	0.033	0.204
AWEI_10	−0.487	0.032	0.030	−0.200
AWEI_11	−0.655	0.012	0.009	−0.241
AWEI_12	−0.600	0.025	0.026	−0.214
AWEI_13	−0.566	−0.058	−0.056	−0.160
AWEI_14	−0.470	−0.009	−0.004	−0.235
AWEI_15	−0.547	0.017	0.018	−0.301
AWEI_16	−0.687	0.045	0.045	−0.196
AWEI_17	−0.798	−0.017	−0.021	−0.211
AWEI_18	−0.641	0.008	0.006	−0.168
AWEI_19	−0.747	−0.042	−0.043	−0.229
Mean	−0.571	−0.009	−0.009	−0.203
Std. Dev	0.105	0.080	0.075	0.029
Minimum	−0.798	−0.331	−0.309	−0.263
Maximum	−0.430	0.050	0.045	−0.155
Mean − Std. Dev	−0.676	−0.089	−0.084	−0.232

**Table 2:** AWEI threshold improvements are based on the mean value calculation of the local threshold of the second-stage split scene image selection for surface water detection.

Approach class threshold calculation	AWEI equation	Threshold improvement value
Equal interval	4 x (G-MIR) − (0.25 x NIR + 2.75 x SWIR)	≥−0.676
Quantile	4 x (G-MIR) − (0.25 x NIR + 2.75 x SWIR)	≥−0.089
Geometrical interval	4 x (G-MIR) − (0.25 x NIR + 2.75 x SWIR)	≥−0.084
Natural break	4 x (G-MIR) − (0.25 x NIR + 2.75 x SWIR)	≥−0.232

**Table 3:** Summary of the classification accuracy assessment of the AWEI threshold improvement.

Class threshold method	Threshold	Land cover class	User Accu. (%)	Product Accu. (%)	Comm. error (%)	Omi. error (%)	Total error (%)	Kappa	Overall Accu. (%)
Normal threshold	≥0.000	Water	99.82	78.07	0.18	21.93	22.11	0.876	98.17
		Nonwater	98.16	99.98	1.84	0.02	1.86		
Equal interval	≥−0.676	Water	27.65	99.99	72.35	0.01	72.36	0.187	49.38
		Nonwater	99.99	37.26	0.01	62.74	62.75		
Quantile	≥−0.089	Water	99.69	98.21	0.31	1.79	2.10	0.987	99.84
		Nonwater	99.87	99.98	0.13	0.02	0.15		
Geometrical interval	≥−0.084	Water	99.70	98.11	0.30	1.89	2.19	0.987	99.84
		Nonwater	99.86	99.98	0.14	0.02	0.16		
Natural break	≥−0.232	Water	98.56	99.67	1.44	0.33	1.77	0.989	99.86
		Nonwater	99.98	99.89	0.02	0.11	0.13		

**Table 4:** Summary of the classification accuracy of the AWEI threshold improvement based on the results of the natural break threshold to detect lake surface water, tested at several locations that could represent other lake characteristics in Indonesia.

Test site	Threshold	Land cover class	User Accu. (%)	Product Accu. (%)	Kappa	Overall Accu. (%)
Maninjau	≥−0.232	Water	97.24	99.41	0.98	99.55
		Nonwater	99.91	99.57		
Singkarak	≥−0.232	Water	97.18	99.74	0.98	99.55
		Nonwater	99.96	99.51		
Kerinci	≥−0.232	Water	95.99	99.72	0.97	99.43
		Nonwater	99.96	99.38		
Rawa Pening	≥−0.232	Water	82.71	87.79	0.73	93.58
		Nonwater	98.40	98.01		
Batur	≥−0.232	Water	96.46	97.75	0.97	99.34
		Nonwater	99.71	99.54		
Sentarum	≥−0.232	Water	87.99	91.92	0.79	94.33
		Nonwater	99.61	99.39		
Jempang	≥−0.232	Water	83.85	76.66	0.76	93.83
		Nonwater	95.56	97.15		
Tempe	≥−0.232	Water	77.98	99.37	0.85	95.77
		Nonwater	99.88	95.14		
Matano	≥−0.232	Water	97.68	99.18	0.98	99.44
		Nonwater	99.82	99.49		
Poso	≥−0.232	Water	98.77	99.56	0.98	99.59
		Nonwater	99.86	99.59		
Limboto	≥−0.232	Water	91.16	93.26	0.91	98.23
		Nonwater	99.13	98.84		
Tondano	≥−0.232	Water	94.97	98.51	0.96	98.86
		Nonwater	99.69	98.93		
Sentani	≥−0.232	Water	97.19	92.39	0.94	98.20
		Nonwater	98.41	99.43		
Rawa Danau	≥−0.232	Water	-	-	-	-
		Nonwater	-	-	-	-

## Data Availability

Landsat 8 was performed using the Google Earth Engine (GEE) platform. Input data were obtained based on Landsat 8 Surface Reflectance Tier 1 data collection. This data set comprises the atmospherically corrected surface reflectance, which is based on the Landsat Ecosystem Disturbance Adaptive Processing System (LaRSC), and the various stages of the process consisting of cloud, shadow, water, and snow mask are produced using CFMASK. Detailed technical information and explanations regarding the data can be accessed at https://developers.google.com/earth-engine/datasets/catalog/LANDSAT_LC08_C01_T1_SR.
